# A comparison of human skeletal muscle cell maturation in 2D versus 3D culture: A quantitative proteomic study

**DOI:** 10.14814/phy2.70420

**Published:** 2025-06-13

**Authors:** Benjamin R. Tollitt, Samantha W. Jones, Jessica Ohana, James R. Henstock, Malcolm J. Jackson, Anne McArdle

**Affiliations:** ^1^ MRC‐Versus Arthritis Centre for Integrated Research into Musculoskeletal Ageing (CIMA), Department of Musculoskeletal and Ageing Science, Institute of Life Course and Medical Sciences University of Liverpool Liverpool UK; ^2^ MyoLine Platform, Sorbonne Université, Inserm Institut de Myologie, Centre de Recherche en Myologie Paris France

**Keywords:** bioengineering, hydrogel, maturation, proteomics, skeletal muscle

## Abstract

Compared with 2D monolayers, 3D models more closely mimic native muscle tissue and allow functional measurements. A more complete understanding of how culture conditions and duration affect myotube maturity/function is crucial for validating the transition to 3D systems. Human skeletal muscle cells were cultured as 2D monolayers or within 3D hydrogels for up to 21 days. Quantitative proteomic analysis and functional measurements were conducted to evaluate muscle cell differentiation. Myoblasts differentiated into myotubes by 8 days in both environments; however, at Day 8, 3D constructs exhibited a predominantly slow‐twitch phenotype, compared with the mixed fiber type of 2D monolayers. By Day 21, 3D constructs demonstrated enhanced mitochondrial maturity, extracellular matrix remodeling, and a fast‐twitch phenotype, indicated by increased myosin‐2 abundance (Log2(FC)>1.29, *p* <0.05). Passive tension increased by >20% following prolonged culture of 3D muscle constructs, but contractile forces reduced by >40%. This study provides a comprehensive proteomic profile of human skeletal muscle cells in 2D and 3D, demonstrating that 3D culture promoted myotube maturity and highlighting the importance of selecting appropriate culture conditions. Data suggest 8 days of differentiation as ideal for achieving peak contractile force in 3D constructs, providing optimal models for testing interventions aimed at preserving muscle function.

## 1 INTRODUCTION

Muscle contraction is an essential physiological process enabling movement to perform daily activities and to maintain posture and balance. However, musculoskeletal diseases, such as sarcopenia, muscular dystrophy, and cancer cachexia, result in the loss of this key function, severely impacting quality of life (Argilés et al., 2014; Dennison et al., 2017; Duan et al., 2021; Young et al., 2021). Understanding the pathophysiology of these diseases and developing effective treatments remain a significant challenge, in part due to limitations in the available in vitro and in vivo models of human skeletal muscle.

In vitro research has predominantly relied on two‐dimensional (2D) cultures of mammalian muscle cells, driven to differentiate from myoblasts into myotubes adhered to flat culture surfaces. These monolayers typically lack the myotube maturity, complex alignment, and spatial organization seen in native muscle tissue and fail to fully recapitulate the complexity of interactions between myotubes and the extracellular matrix (ECM) or the properties to enable functional measures of contractile force (Carraro et al., 2022; de Jong et al., 2024; Dessauge et al., 2021; Resnicow et al., 2010; Suetterlin et al., 2022). Alternatively, in vivo or ex vivo animal skeletal muscle tissue, typically from rodents, is used. However, rodent tissue may not be an appropriate model in certain situations, given genetic, biochemical, and physiological differences in fiber type between humans and rodents (Guo et al., 2014; Mathewson et al., 2012). These limitations underscore the importance of human‐specific models of mature muscle to accurately investigate physiology.

To address this gap, the development of three‐dimensional (3D) in vitro bioengineered skeletal muscle models, often referred to as muscle constructs, allows for the culture and differentiation of human myoblasts into myotubes within an environment mimicking the structure and function of native muscle ECM (Dessauge et al., 2021; Duval et al., 2017). A common approach used is to encapsulate the cells within a 3D scaffold or hydrogel to promote myotube alignment and enable functional measurements of contraction to be undertaken on myotubes with a physiologically relevant genotype. Furthermore, the use of 3D bioengineered models as alternatives to animal models helps to alleviate ethical concerns, while also reducing the high costs and time‐consuming nature of animal‐based research (Alonso‐Puyo et al., 2024; Mehmood et al., 2024). While these 3D models hold significant promise, there are still gaps in our understanding of how culturing cells in either 2D monolayers or 3D environments impacts the biochemistry and contractile function of the resulting myotubes. In particular, the relationship between culture condition, duration, and myotube function remains poorly characterized.

Previous studies have measured biophysical and biomechanical properties of 3D muscle constructs, such as stiffness, elasticity, passive tension, and force of contraction, with some studies assessing changes to these parameters with varying duration in culture (Furuhashi et al., 2021; Hofemeier et al., 2021; Vesga‐Castro et al., 2022). However, many of these muscle construct models use cells of non‐human origin, reducing physiological relevance for studying human muscle disorders. For cells of human origin, either primary cell isolates, induced pluripotent stem cells (iPSCs) or an immortalized cell line can be used, although each brings its own advantages and limitations. Many studies have used primary cells and iPSCs to produce 3D muscle constructs, but these raise complications due to availability and difficulty of handling (Maffioletti et al., 2018; van der Wal et al., 2023; Wroblewski et al., 2024). While immortalized cell lines derive from a single point of origin, they offer the unique advantages of ease of access and greater consistency across passages and between laboratories. The AB1167 cell line is an accepted immortalized human cell line with a robust phenotype, repeatedly used as a healthy control in previous studies (Cea et al., 2020; Lad et al., 2024; Nguyen et al., 2021). Additionally, previous studies often fail to link functional changes observed to biochemical changes in the myotubes, restricting identification of potential targets within the myotubes for investigating disease progression or developing therapeutic intervention to improve muscle function. Proteomic analysis offers a powerful solution by providing a comprehensive profile of the proteome of myotubes, enabling global analysis of how these biochemical properties change under different conditions.

Many studies utilizing proteomics to assess cell development have focused on the effects of culturing cancer cell lines in 3D compared with 2D (Guo et al., 2024; Kaczmarczyk et al., 2021; Zhang et al., 2024). A number of these studies report changes in the metabolic profile of human cancer cell lines when cultured in 3D as spheroids compared to the same cell lines cultured as 2D monolayers. Data show protein changes associated with increased oxidative phosphorylation, glycolysis, and gluconeogenesis pathways in 3D samples (He et al., 2014; Yue et al., 2016). Similar findings of elevated metabolic pathways including glycolysis, gluconeogenesis, and the citric acid cycle have also been reported in mouse adipocyte cells encapsulated in 3D hydrogels compared with 2D monolayers (Lee et al., 2019). Proteomic studies have also reported ECM remodeling and reorganization in both human skin fibroblasts, cultured in a 3D collagen‐based hydrogel, and human hepatocellular carcinoma cells, cultured as spheroids, compared to 2D monolayers (Hurrell et al., 2019; Tölle et al., 2018). Despite extensive research comparing 2D and 3D culture for various cell types, there is a notable lack of such studies for human skeletal muscle myotubes, where 3D culture is of particular importance to potentially enable additional maturation and contractile force measurements.

The aim of the present study was to combine functional measures of human myotubes cultured in a 3D environment with quantitative proteomic analysis of myotubes in both 2D and 3D environments at different time points to obtain a powerful insight into how both culture conditions and duration affect the biochemistry and maturity of myotubes. Furthermore, this validation of the transition from 2D to 3D in vitro models of human skeletal muscle is a crucial step in developing a platform for studying human skeletal muscle disorders and assessing therapeutic interventions.

## 2 MATERIALS AND METHODS

### 2.1 Culture of immortalized human myoblasts

The immortalized human skeletal muscle cell line (Lot AB1167) derived from the *Fascia lata* of a 20‐year‐old male was used in this study and was provided by MyoLine, the platform for immortalisation of human cells from the Centre of Research in Myology (Mamchaoui et al., 2011). This cell line differentiates to produce myotubes that contract spontaneously and following electrical stimulation. Proliferating myoblasts were split using standard methods at 70% confluency and cultured in complete growth medium consisting of Skeletal Muscle Basal Medium supplemented with 50 μg/mL bovine fetuin, 10 ng/mL human epidermal growth factor, 1 ng/mL human basic fibroblast growth factor, 10 μg/mL human insulin, 0.4 μg/mL dexamethasone (all contained in C‐23060; PromoCell, Heidelberg, Germany), 20% (v/v) foetal bovine serum (FBS; 16140071; Gibco, Waltham, MA, USA), 2 mM L‐glutamine (59202C; Gibco, Waltham, MA, USA) and 10 μg/mL gentamicin (G1397; Sigma‐Aldrich, St. Louis, MO, USA). Cells were incubated at 37°C, 5% CO_2_ in a humidified atmosphere, with medium changed every 48 hours.

Differentiation into two‐dimensional (2D) myotube monolayers was induced by replacing complete growth medium with complete differentiation medium consisting of Dulbecco's Modified Eagle's Medium (DMEM), with high glucose, GlutaMAX™ supplement without sodium pyruvate (61965026; Gibco, Waltham, MA, USA), supplemented with 2% (v/v) horse serum (H1270; Gibco, Waltham, MA, USA), 10 μg/mL recombinant human insulin (91077C; Sigma‐Aldrich, St. Louis, MO, USA) and 10 μg/mL gentamicin, with medium changed every 48 h.

### 2.2 Fabrication of three‐dimensional (3D) human muscle constructs

**FIGURE 1:**
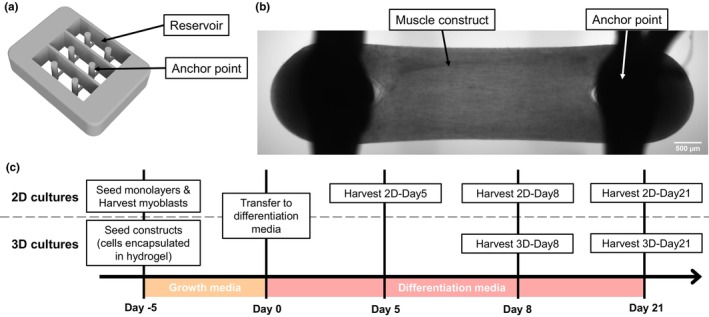
Experimental timeline and scaffold design for 3D culture. (a) Computer Aided Design (CAD) rendering of the scaffold used to culture 3D muscle constructs. The cell/hydrogel mixture was dispensed into the reservoir and muscle constructs self‐assembled around the two anchor points. (b) Representative brightfield image of a whole muscle construct after 8 days of differentiation, scale bar 500 μm. (c) Timeline of the experimental protocol showing steps for cell seeding and sample harvesting for both 2D and 3D culture.

Custom scaffolds were 3D printed using an UltiMaker 2+ using polylactic acid (PLA) filament (Figure 1a). These scaffolds comprised three reservoirs, each with a pair of anchor points 5 mm apart around which muscle constructs can self‐assemble, producing muscle constructs of 5 mm in length. Scaffolds were sterilized in 70% ethanol, then submerged in sterile 0.2% (w/v) Pluronics™ F127 (Sigma‐Aldrich, St. Louis, MO, USA) overnight at 4°C before being embedded in a thin layer of 4% agarose (Sigma‐Aldrich, St. Louis, MO, USA) to prevent the myoblast/hydrogel mixture from leaking.

Myoblasts were dissociated from culture flasks using 1X Trypsin–EDTA (Gibco, Waltham, MA, USA) and the appropriate number of myoblasts were encapsulated in sufficient hydrogel solution to prepare three constructs, filling one scaffold. For a single muscle construct, 150 μL of hydrogel solution was prepared consisting of 520 μg/mL bovine plasma fibrinogen (F8630; Merck, Darmstadt, Germany), 20% (v/v) growth‐factor reduced ECM Gel from Engelbreth‐Holm‐Swarm murine sarcoma (E6909; Sigma‐Aldrich, St. Louis, MO, USA), 2.2 U/mL bovine thrombin (T4648; Sigma‐Aldrich, St. Louis, MO, USA) and 2.5 × 10^5^ myoblasts in complete growth medium (described above) supplemented with 1.5 mg/mL aminocaproic acid (ACA; Sigma‐Aldrich, St. Louis, MO, USA).

The cell‐laden hydrogel mixture was dispensed into the scaffold reservoirs and allowed to polymerize at 37°C, 5% CO_2_ for 15 min before being incubated in complete growth medium supplemented with 1.5 mg/mL ACA. After 5 days in growth medium, with the medium changed every 48 h, muscle constructs spontaneously assembled around the two anchor points (Figure 1b). Muscle constructs were then transferred to complete differentiation medium (described above) supplemented with 2 mg/mL ACA, with the medium changed every 48 h for a total time described below.

### 2.3 Sample collection

Figure 1c displays the experimental timeline for the growth and collection of both 2D and 3D culture samples. For analysis of myoblasts, cells were harvested from 2D culture during their active proliferation phase (*n* = 5).

When differentiated in 2D monolayers, the immortalized human skeletal muscle cell line typically forms networks of myotubes that spontaneously contract and detach from the culture surface around Day 6 of differentiation, which are then removed during media changes. To assess whether proteomic differences exist in 2D samples before and after the loss of the actively contractile layer, 2D samples were harvested at Day 5 of differentiation (2D‐Day5; *n* = 5) and Day 8 of differentiation (2D‐Day8; *n* = 5). Myotubes were also harvested after an extended differentiation period of 21 days (2D‐Day21; *n* = 5). All cells were harvested by washing in phosphate‐buffered saline (PBS), before scraping into PBS and centrifuging at 200×*g* for 5 min. Supernatant was removed and resultant cell pellets were snap‐frozen in LN2 and stored at −80°C until further processing.

To temporarily align with the differentiation of 2D monolayers, samples for 3D constructs were also harvested at 8 days following the initiation of differentiation (3D‐Day8; *n* = 5) and after extended differentiation of 21 days (3D‐Day21; *n* = 5). Constructs were harvested by rinsing in PBS, snap‐freezing in liquid nitrogen, and storing at −80°C until processed.

### 2.4 Protein extraction and digestion for proteomic analysis

Protein extraction was performed by adding 100 μL lysis buffer (1% SDS, 1% Igepal, 1% NA‐Deoxycholate, 125 mM NaCl, 5 mM EDTA, 100 mM Tris pH 8, 1X phosSTOP [4906845001; Sigma‐Aldrich, St. Louis, MO, USA], 1X cOmplete protease inhibitor [11873580001; Sigma‐Aldrich, St. Louis, MO, USA]) to the cell pellet or construct, which was then sonicated for 6 cycles of 30 s in a Diagenode Bioruptor. The sample was then centrifuged at 13,000×*g* for 15 min at 4°C, and the supernatant retained. Protein quantification was determined using a Pierce BCA Protein Assay Kit (Thermo Fisher Scientific, Waltham, MA, USA) with bovine serum albumin (BSA) as a protein standard.

For digestion, 4 μg of protein was diluted in a final volume of 85 μL using 25 mM Ambic. Cysteine reduction was performed using dithiothreitol (DTT) (60°C, 10 min, 4 mM) and alkylated with iodoacetamide (room temp, 30 min, 14 mM). SeraMag™ beads were added at a 10:1 bead‐to‐protein ratio, and acetonitrile was added at >80% (v/v) and incubated at 75×*g*, 20°C for 30 min. Supernatant was removed using a magnetic rack, and beads were washed twice with 100% acetonitrile, then once with 80% acetonitrile/20% High‐performance liquid chromatography (HPLC) water mix. Following vacuum drying for 10 min, beads were resuspended in 98 μL of 25 mM Ambic and sonicated for 5 min. A total of 0.1 μg Trypsin was added and incubated at 38 × g, 37°C for 16 h. Peptide solution was collected in Lobind tubes using a magnetic rack, and samples were incubated at 37°C for 45 min with trifluoroacetic acid (TFA) at a final concentration of 0.5% (v/v). Samples were left at 4°C for 30 min, then vacuum dried to completion and resuspended in 97% LCMS H_2_O/3% acetonitrile/1% TFA. Following 10 min sonication, samples were centrifuged at 13,000×*g* for 15 min at 4°C, and the resulting supernatant was used for liquid chromatography tandem mass spectrometry (LC–MS) analysis.

### 2.5 LC–MS analysis

Analysis was performed on a NanoElute chromatography system (Bruker Daltonics) coupled to a timsTOF HT mass spectrometer (Bruker Daltonics) with a CaptiveSpray ion source (Bruker Daltonics). The sample (1 μL, 200 ng) was loaded onto the trapping column (Thermo Trap Cartridge, PepMap100, C18, 300 μm × 5 mm) and resolved on the analytical column (PepSep, twenty‐five Series 150 μm, 1.5 μm column) at 35°C using a gradient of 98% A (0.1% formic acid)/2% B (acetonitrile, 0.1% formic acid), increasing to 65% A/35% B over 60 min at a flow rate of 0.5 μL/min, followed by a wash step of 95% B. Acquisition was performed in data‐independent acquisition (DIA) parallel accumulation‐serial fragmentation (PASEF) mode, defined with a m/z range of 475–1000 and a mobility range of 1/K0 = 0.85–1.3 Vs/cm^2^ using equal ion accumulation and ramp times of 100 ms in the dual TIMS analyzer. Collision energy was lowered as a function of increasing ion mobility from 59 eV at 1/K0 = 1.4 Vs/cm^2^ to 20 eV at 1/0 = 0.6 Vs/cm^2^. The dia‐PASEF window scheme consisted of 21 isolated windows of 25 m/z width with no mass overlap and 1 ion mobility window. All samples were analyzed in random order.

### 2.6 Data quality check

Raw data files were searched against the UniProt Homo Sapiens database (20,383 entries) and a contaminant database (116 sequences, 38,459 residues) using Spectronaut Direct DIA+ with the following search parameters: variable modification of methionine oxidation and acetyl (Protein N‐term), a fixed cysteine carbamidomethylation modification, limited to 2 missing cleavages, and false discovery rate (FDR) set to 1%. As the 3D muscle construct samples contain proteins from both human and non‐human origin, a proteotypic filter was applied to include only proteins of confirmed human origin, ensuring the quantified dataset was localized to the human myotubes and excreted proteins, not the exogenous proteins from the supporting hydrogel in 3D culture samples.

### 2.7 Proteomic data analysis

All data processing was carried out using R version 4.3.1. Proteins with more than 7% missing values across the 30 samples were excluded before imputation was performed using the k‐nearest neighbor algorithm (*k* = 10) (Troyanskaya et al., 2001). Variance stabilizing normalization (VSN) and differential abundance analysis were carried out using the NormalyzerDE package (v. 1.18.1), applying limma statistical comparisons and adjusting for the FDR using Benjamini‐Hochberg correction (Benjamini & Hochberg, 1995; Willforss et al., 2019).

Proteins were identified as differentially abundant proteins (DAPs) if the FDR adjusted *p*‐value (pAdj) was *p* Adj ≤0.05 and the fold change (FC) was FC > |1|. Enrichment analysis was performed on differentially abundant proteins using the clusterProfiler package (v. 4.8.3) for Kyoto Encyclopedia of Genes and Genomes (KEGG) pathway enrichment analysis, with Human as the reference organism (Yu et al., 2012). Enriched canonical pathways were identified using Ingenuity Pathway Analysis (IPA) software (IPA, Qiagen Redwood City, USA).

All figures shown were created using the R package ggplot2 (v. 3.4.3) or QIAGEN IPA.

### 2.8 Muscle construct contraction protocol

Functional measures of muscle constructs were taken for 3D‐Day8 (*n* = 8) and 3D‐Day21 (*n* = 12) sample groups. In order to determine the optimal length of the muscle construct that elicited the maximum contractile force, the length of each construct was increased and contractile force measured every 2 min until maximum contractile force was achieved in a similar manner to ex vivo studies using a custom dual‐axis micropositioner stage (Koh et al., 2003; Paul et al., 2023; Vasilaki et al., 2016). Preliminary data suggested that lengthening to above 9 mm, corresponding to 180% of the original culture length (5 mm), resulted in the initiation of construct failure in a sub‐group and so 8 mm was set as the maximum length used. After carefully removing the individual muscle constructs from the scaffold, constructs were attached to a custom 3D printed reservoir filled (volume of 0.375 mL) with complete differentiation medium supplemented with 2 mg/mL ACA. Muscle constructs were lengthened in 10% increments of their initial culture length (5 mm), with passive tension and tetanic force generation measured at each lengthening step, until a final position of 160% of initial length. Passive tension and contractile force following electrical stimulation was recorded using a strain gauge (SI‐KG7A, World Precision Instruments) with a Grass S88 Stimulator and custom‐made amplifier. Electrical stimulation occurred via stainless steel electrodes (50 Hz, pulse duration of 2 ms for a train duration of 500 ms at 7 V). Each muscle construct was electrically stimulated to contract three times at each length, with 2 min rest between stimulations, and the maximum tetanic force achieved at each position was recorded.

### 2.9 Immunohistochemistry

After the relevant differentiation period, 2D monolayers of cells (*n* = 6 each) were washed with PBS and fixed in 4% PFA for 10 min at room temperature. Fixed cells were incubated in blocking buffer (PBS with 10% goat serum [Sigma‐Aldrich, St. Louis, MO, USA] and 0.5% Triton X‐100 [Sigma‐Aldrich, St. Louis, MO, USA]) for 1 h at room temperature, then anti‐sarcomeric alpha actinin (1:200 dilution; ab137346; Abcam, Cambridge, UK) diluted in blocking buffer overnight at 4°C. This was removed and cells were rinsed in wash buffer (PBS with 0.2% Tween 20® (Sigma‐Aldrich, St. Louis, MO, USA) and 0.5% Triton X‐100), then incubated for 1 h at room temperature in Alexa Fluor™ 488 goat anti‐mouse secondary antibody (1:500 dilution; A‐11001; Thermo Fisher, Waltham, MA, USA) and DAPI (1:1000 dilution; Thermo Fisher Scientific, Waltham, MA, USA) diluted in wash buffer. After rinsing in wash buffer, images were captured on the Zeiss Observer Apotome microscope and myotube diameter was measured using FIJI ImageJ software (NIH, Bethesda, MD, USA).

A separate set of 3D muscle constructs to those electrically stimulated was harvested at the same timepoints (Day 8, *n* = 4; Day 21, *n* = 5). Muscle constructs were fixed in 4% PFA for 4 h at room temperature, embedded in OCT, and frozen in isopentane cooled with liquid nitrogen. Samples were transferred to a cryostat (Leica 1860) and 15 μm transverse sections were taken from the midpoint of the construct. Cryosections were rehydrated using PBS, then stained with Alexa Fluor™ 388 conjugated phalloidin (1:40 dilution; A12379; Thermo Fisher Scientific, Waltham, MA, USA) and DAPI (1:1000 dilution) for 1 h at room temperature. Sections were rinsed in wash buffer, then mounted with ProLong Gold Antifade (Thermo Fisher Scientific, Waltham, MA, USA) and images were captured on a Zeiss LSM 800 confocal microscope at 20× magnification. Quantification of cryosection images was performed by applying thresholding of the phalloidin staining, then measuring cross‐sectional area following dilation and erosion cycles using ImageJ software.

### 2.10 Statistics

For all “non‐omics” data, normality was assessed with the Shaprio‐Wilk test. For normally distributed data, a parametric unpaired t‐test was performed, for non‐parametric data, the Mann–Whitney test was performed. All statistical analysis was performed on GraphPad Prism 10.1.2.

The mass spectrometry proteomics data have been deposited to the ProteomeXchange Consortium via the PRIDE (Perez‐Riverol et al., 2025) partner repository with the dataset identifier PXD061227.

## 3 RESULTS

**FIGURE 2:**
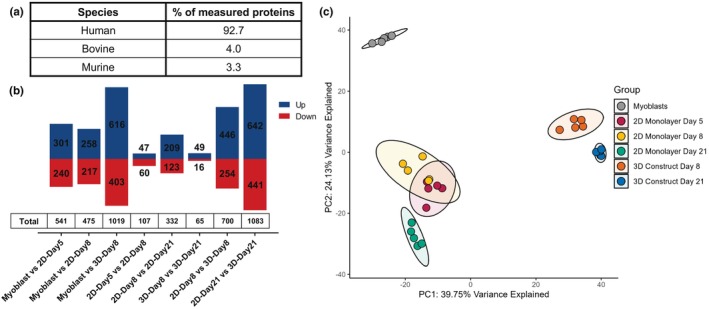
Quantitative proteomic analysis of human skeletal muscle cells at various culture durations in either 2D monolayers or encapsulated in a 3D hydrogel. (a) Species distribution of the number of proteotypic proteins identified in the dataset. Values represent the percentage of unique proteins detected, categorized by species of origin. (b) Bar graphs showing the number of differentially abundant proteins (DAPs) between group comparisons, identifying the counts of proteins with increased abundance (blue) or decreased abundance (red). (c) Principal component analysis (PCA) of proteomic data shows clustering of samples by culture conditions (*n* = 5 for each group).

Quantitative proteomic analysis was performed on human immortalized skeletal muscle cells cultured under different conditions: 2D monolayers or embedded within a 3D hydrogel as muscle constructs for varying culture durations. In total, 4040 proteotypic proteins were analyzed in the dataset across all samples. The majority of unique prototypic proteins identified across all six sample groups were of human origin, with <8% being of bovine or murine origin from the hydrogel components, particularly the Matrigel (Figure 2a).

Principle component analysis (PCA) was conducted to evaluate overall variance in protein abundance across all six sample groups. The first two principal components account for the majority of variance, 39.8% and 24.1%, respectively. The PCA plot shows a discrete cluster for the myoblast samples and displays clear discrete clustering of samples cultured in either 2D or 3D conditions (Figure 2c). Culture time also resulted in strong sample clustering, particularly of samples at day 21 of culture in both environments, and cluster overlap of the 2D‐Day5 and 2D‐Day8 groups.

Differentially abundant proteins (DAPs) between groups were defined by a log2(FC) > |1| and adjusted *p*‐value <0.05 to represent a doubling or halving of protein abundance. Figure 2b displays the total number of DAPs for each comparison, with the number of proteins with increased abundance presented in blue and proteins with decreased abundance in red.

### 3.1 Effects of extended culture time on the proteomic profile of human skeletal muscle cells cultured in 2D

**FIGURE 3:**
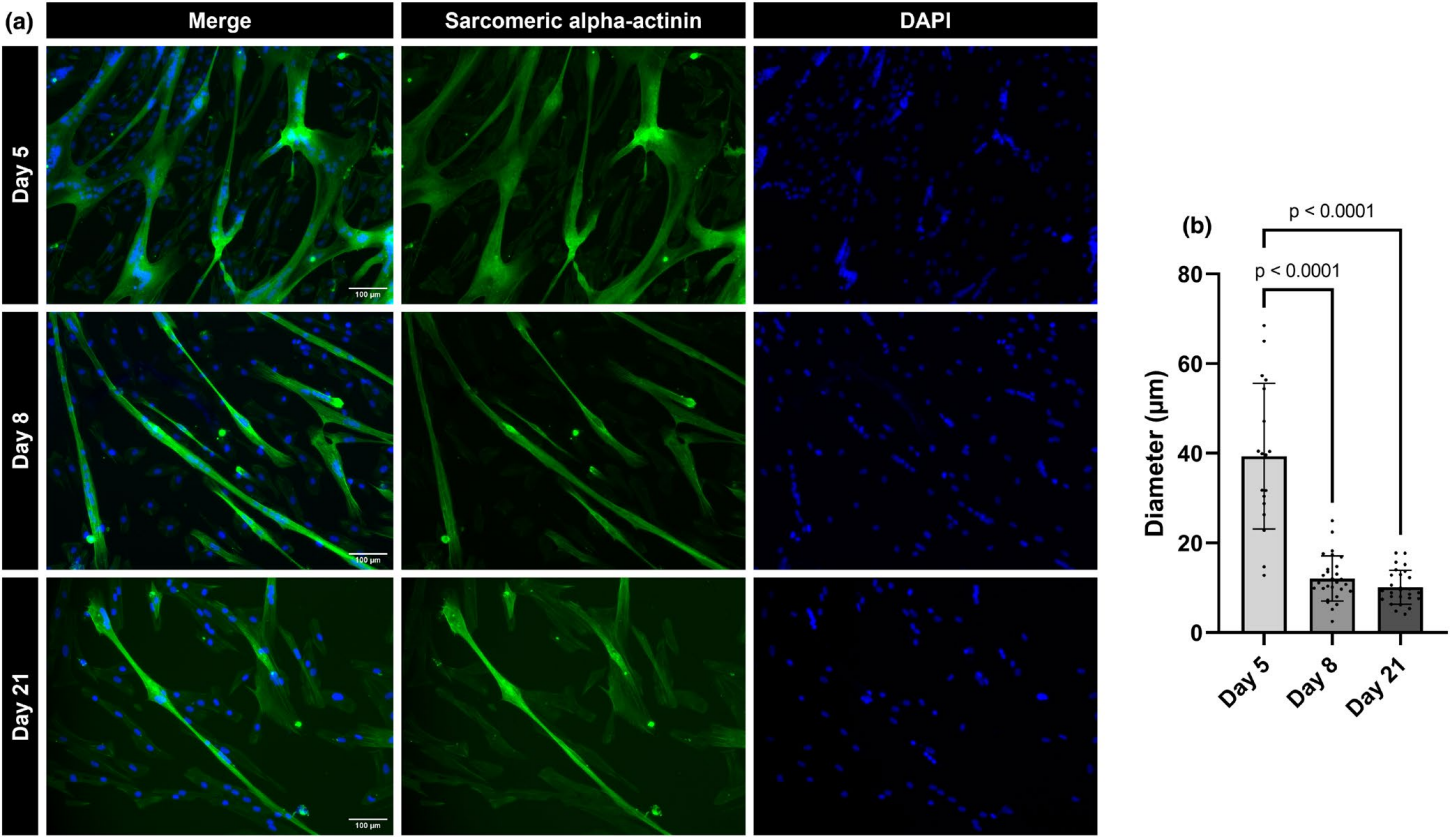
Immunofluorescence staining of human skeletal muscle cells. (a) Representative images of skeletal muscle cells stained after 5, 8, or 21 days of culture in 2D monolayers. Cells were stained for sarcomeric alpha‐actinin and DAPI. Images were captured at 10× magnification. Scale bar 100 μm. (b) Bar chart showing quantification of myotube diameter at each timepoint (*n* = 6 each). Data represents mean ± SD.

Consistent with the results of the PCA plot, comparisons of myoblasts to any other sample group produced a large number of DAPs. A total of 541 DAPs were identified when comparing undifferentiated myoblasts to 2D‐Day5, with 301 up‐regulated and 240 down‐regulated (Figure 2b). While proteomic analysis suggests myoblasts had differentiated into myotubes by day 5 of differentiation, fiber size measurements only stabilized from Day 8 of differentiation (Figure 3).

**FIGURE 4:**
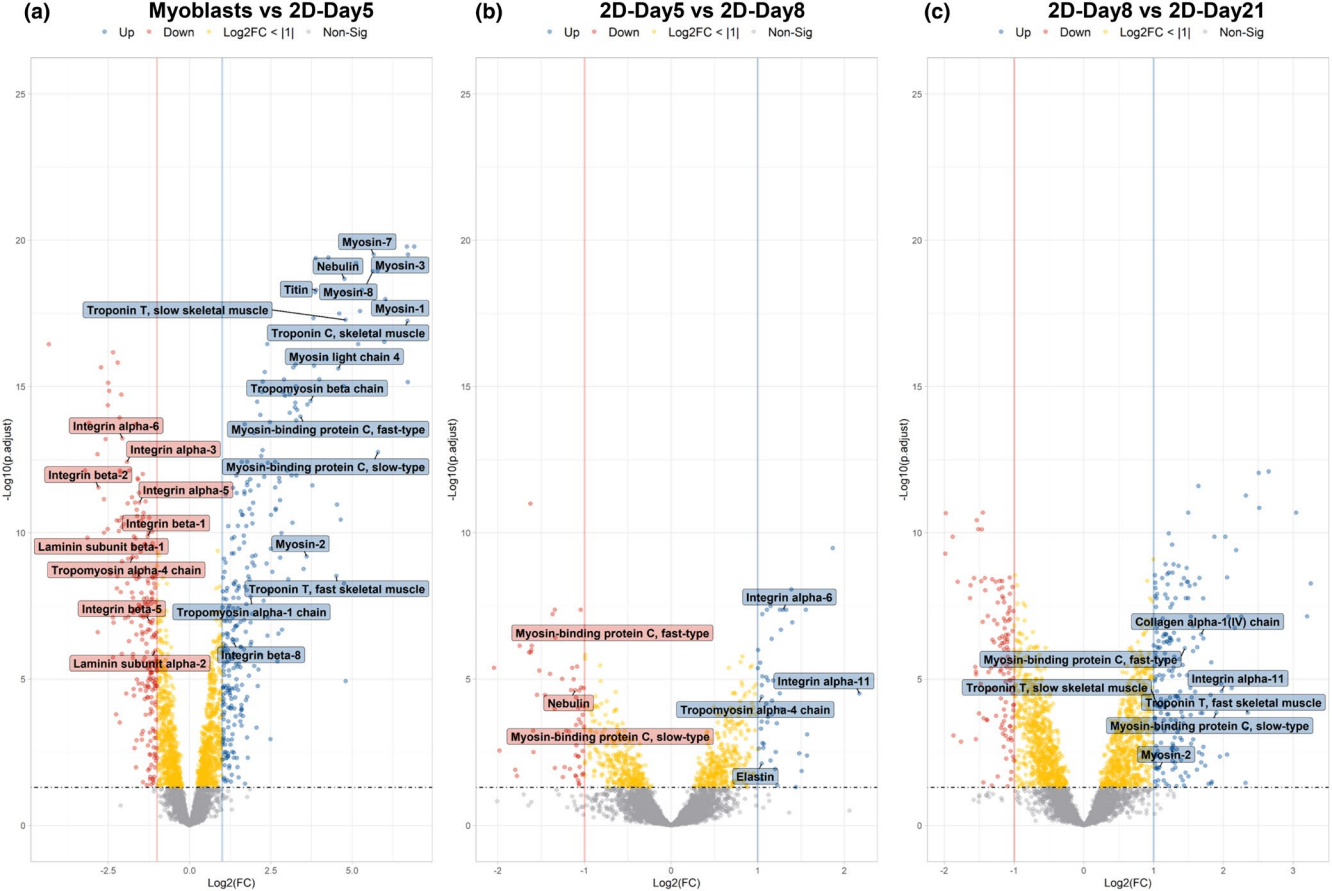
Differential protein abundance in human skeletal muscle cells cultured as 2D monolayers at various stages of differentiation. Volcano plots showing changes in protein abundances of human skeletal muscle cells cultured in 2D monolayers, showing log2 fold change (FC) against –log10 adjusted *p*‐value. Differentially abundant proteins (DAPs) are defined by adjusted *p*‐value <0.05 and log2(FC) > |1|. DAPs are shown in blue (increased abundance) and red (decreased abundance). Proteins with a *p*‐value <0.05 but log2(FC) < |1| are shown in yellow and gray points represent non‐significant proteins. Vertical lines indicate the FC threshold of ±1, and the horizontal dashed line indicates the significance cutoff. Plots show DAPs between (a) myoblasts and Day 5 of differentiation, (b) Day 5 and 8 of differentiation, and (c) Day 8 and 21 of differentiation.

**FIGURE 5:**
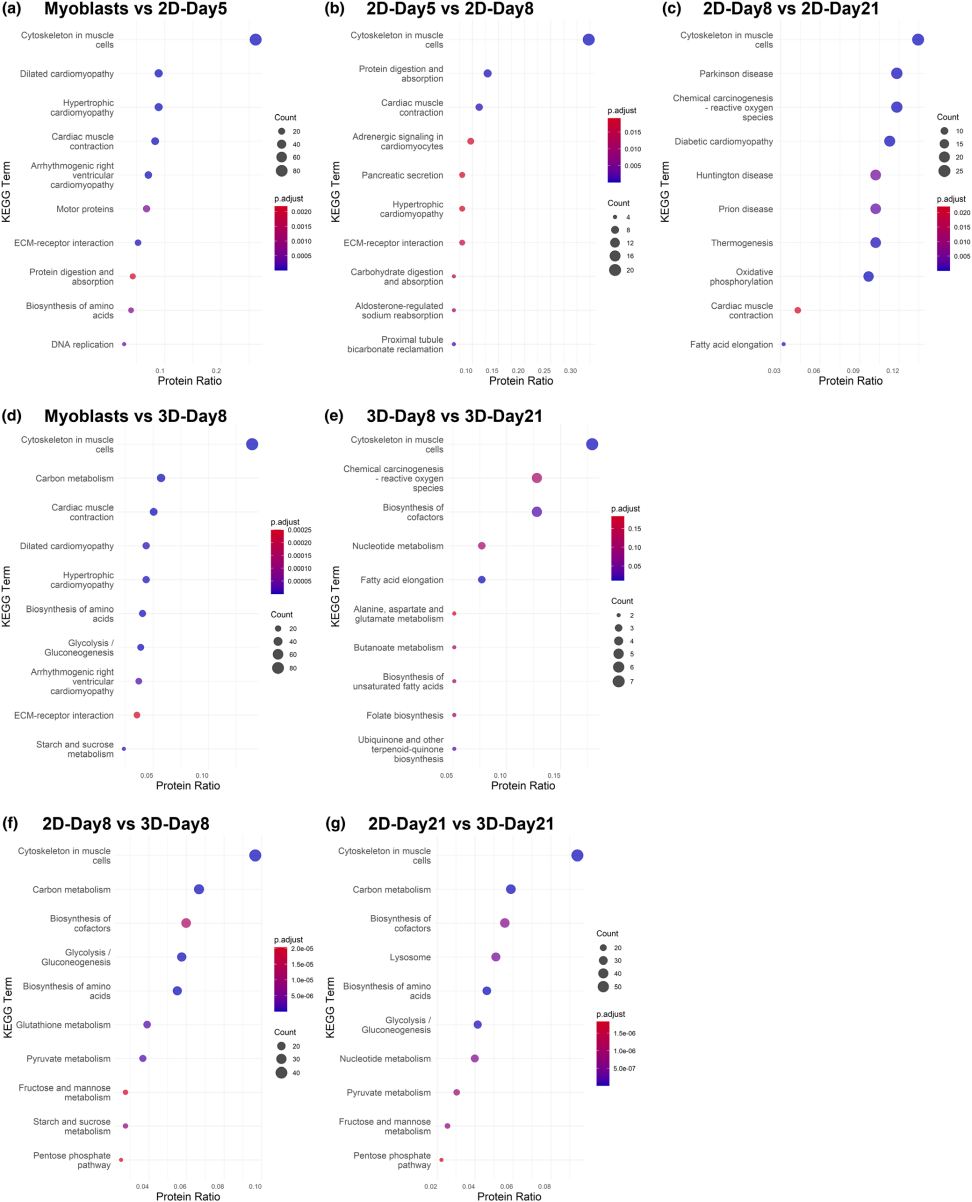
Top enriched KEGG pathways in human skeletal muscle cells in 2D or 3D culture. Top 10 enriched KEGG pathways associated with the differentially abundant proteins (DAPs) between human skeletal muscle cells cultured in various conditions: (a) myoblasts versus Day 5 of differentiation as 2D monolayers, (b) Day 5 versus 8 of differentiation in 2D, (c) Day 8 versus 21 of differentiation in 3D, (d) myoblasts versus Day 8 of differentiation as 3D muscle constructs, (e) Day 8 versus 21 of differentiation in 3D, (f) 2D culture versus 3D culture at Day 8 of differentiation, (g) 2D culture versus 3D culture at Day 21 of differentiation. The *x*‐axis shows the protein ratio (the proportion of identified proteins linked to each pathway), point size indicates the number of proteins mapped to the pathway, and color represents the adjusted *p*‐values.

As anticipated, the volcano plot presented in Figure 4a highlights upregulation of key contractile and cytoskeletal proteins by Day 5 of differentiation, including multiple isoforms of myosin heavy chain (MHC) and troponin, and downregulation of several integrin and laminin isoforms. Culture in 2D monolayers produced myotubes with an approximately equal increase in abundance of embryonic MHC 3, perinatal MHC 8, slow‐twitch (type I) MHC 7, glycolytic fast‐twitch (type IIx) MHC 1, and oxidative fast‐twitch (type IIa) MHC2 (Figure 4a). KEGG pathway analysis revealed that the top enriched pathways were primarily associated with cellular structure, contraction, and extracellular matrix (ECM)‐receptor interactions (Figure 5a).

Also correlating with the PCA results, only a small number of DAPs were found when comparing 2D‐Day8 to 2D‐Day5 (107 DAPs). Integrin isoforms were present among the 47 proteins with increased abundance in 2D‐Day8 compared with 2D‐Day5, and both fast‐ and slow‐type myosin‐binding protein C among the 60 proteins with decreased abundance (Figure 4b). However, the remaining 2D‐Day8 myotubes showed a significantly narrower myotube diameter than 2D‐Day5 (Figure 3). These data suggest that myotube maturation had primarily occurred by 5 days in differentiation medium, and a largely similar proteomic profile existed before and after the loss of the actively contractile myotube layer.

The 332 DAPs identified when comparing 2D‐Day8 and 2D‐Day21 are represented on the volcano plot in Figure 4c. The increased abundance of both fast and slow isoforms of myosin‐binding protein C and Troponin T shows that the mixed phenotype of muscle fiber types was maintained. Enriched KEGG pathway analysis for this comparison revealed changes in the cytoskeleton of muscle cells, as well as energy metabolism, in particular oxidative phosphorylation (Figure 5c). The IPA analysis also showed that the top enriched canonical pathways were associated with oxidative phosphorylation and changes to the mitochondria to improve energy metabolism, with increased abundance of subunits that compose the mitochondrial electron transport chain (ETC), in particular subunits of complex I and ATP synthase (Table 1). These data suggest a second phase of maturation potentially associated particularly with mitochondrial changes/development.

**TABLE 1:** Top up‐ and down‐regulated canonical pathways of human skeletal muscle cells differentiated as 2D monolayers for 8 versus 21 days.

	Canonical pathway	*z*‐score	Ratio	−Log(*p*‐value)	Proteins present in dataset
Up‐regulated	Oxidative phosphorylation	4.36	0.17	15.00	↑ATP5F1A, ↑ATP5F1D, ↑ATP5ME, ↑ATP5MG, ↑ATP5PD, ↑ATP5PF, ↑ATP5PO, ↑COX7A1, ↑CYB5A, ↑CYC1, ↑CYCS, ↑DMAC2L, ↑NDUFB6, ↑NDUFS4, ↑NDUFS7, ↑NDUFV3, ↑UQCR10, ↑UQCRH, ↑UQCRQ
	Electron transport, ATP synthesis, and heat production by uncoupling proteins	4.36	0.15	13.90	↑ATP5F1A, ↑ATP5F1D, ↑ATP5ME, ↑ATP5MG, ↑ATP5PD, ↑ATP5PF, ↑ATP5PO, ↑CYC1, ↑CYCS, ↑DMAC2L, ↑ETFDH, ↑NDUFAF1, ↑NDUFB6, ↑NDUFS4, ↑NDUFS7, ↑NDUFV3, ↑UQCR10, ↑UQCRH, ↑UQCRQ
	Cristae formation	3.46	0.39	14.40	↑APOO, ↑APOOL, ↑ATP5F1A, ↑ATP5F1D, ↑ATP5ME, ↑ATP5MG, ↑ATP5PD, ↑ATP5PF, ↑ATP5PO, ↑DMAC2L, ↑DNAJC11, ↑TMEM11
	Mitochondrial protein import	3.16	0.16	7.81	↑ATP5F1A, ↑CHCHD4, ↑CYC1, ↑DNAJC19, ↑PAM16, ↑SLC25A12, ↑SLC25A4, ↑TIMM13, ↑TOMM40, ↑VDAC1
	Neutrophil extracellular trap signaling pathway	3.13	0.05	6.14	↑ATP5F1A, ↑ATP5F1D, ↓CERT1, ↓COL3A1, ↑COL4A1, ↑CYC1, ↑NDUFAF1, ↑NDUFB6, ↑NDUFS4, ↑NDUFS7, ↑NDUFV3, ↑PAM16, ↑PLCD3, ↓PRKD1, ↓RIPK1, ↑SLC25A4, ↑TIMM13, ↑TOMM40, ↑TSPO, ↑VDAC1
Down‐regulated	Mitochondrial dysfunction	−4.16	0.08	13.40	↑ATP5F1A, ↑ATP5F1D, ↑ATP5ME, ↑ATP5MG, ↑ATP5PD, ↑ATP5PF, ↑ATP5PO, ↑COX7A1, ↑CYB5A, ↑CYC1, ↑CYCS, ↑DLAT, ↑DMAC2L, ↑HTRA2, ↑IDH2, ↑MGST3, ↑NDUFAF1, ↑NDUFB6, ↑NDUFS4, ↑NDUFS7, ↑NDUFV3, ↑OPA1, ↑SOD2, ↑TOMM40, ↑UQCR10, ↑UQCRH, ↑UQCRQ, ↑VDAC1
	Protein folding	−2.45	0.06	2.65	↓CCT2, ↓CCT5, ↓CCT6B, ↓CCT8, ↓PFDN5, ↓USP11
	RHOA Signaling	−2.45	0.05	2.15	↓ARHGEF1, ↓LPAR1, ↑MYLK, ↓PFN1, ↓SEPTIN10, ↓SEPTIN9
	Purine nucleotides de novo biosynthesis II	−2.00	0.36	4.98	↓GART, ↓IMPDH2, ↓PAICS, ↓PFAS
	Parkinson's signaling pathway	−1.90	0.03	2.01	↓ALDH1A1, ↑ALDH5A1, ↑ATP6AP1, ↑NDUFAF1, ↑NDUFB6, ↑NDUFS4, ↑NDUFS7, ↑NDUFV3, ↑SLC11A2, ↑UCHL1

*Note*: Top 5 up‐ and down‐regulated canonical pathways, ranked based on *z*‐score, identified by IPA analysis. For each pathway, the *z*‐score, ratio (proportion of proteins involved in the pathway), and −log(*p*‐value) are provided. The list of proteins from the dataset contributing to the differential expression of each pathway is included, with proteins showing increased abundance represented with up‐arrows and proteins showing decreased abundance represented with down‐arrows.

### 3.2 Effects of extended culture time on the proteomic profile of human skeletal muscle cells cultured in 3D hydrogels as muscle constructs

**FIGURE 6:**
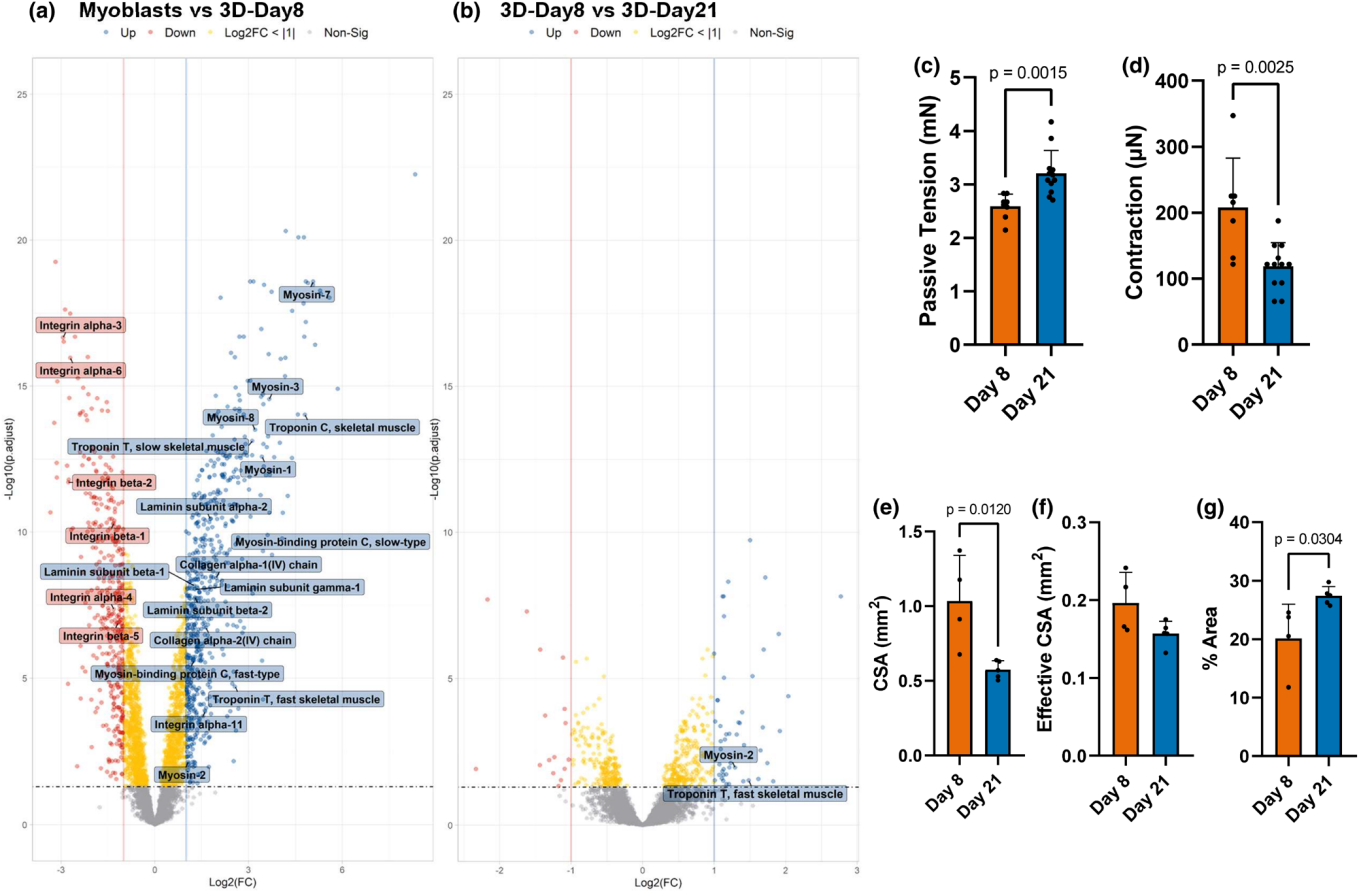
Differential protein abundance and changes in functional measures of human skeletal muscle cells cultured as 3D muscle constructs at different stages of differentiation. (a, b) Volcano plots showing changes in protein abundance of human skeletal muscle cells cultured in 2D as myoblasts or encapsulated in a 3D hydrogel, showing log2 fold change (FC) against –log10 adjusted *p*‐value. Differentially abundant proteins (DAPs) are defined by adjusted *p*‐value <0.05 and log2(FC) > |1|. DAPs are shown in blue (increased abundance) and red (decreased abundance). Proteins with a *p*‐value <0.05 but log2(FC) < |1| are shown in yellow and gray points represent non‐significant proteins. Vertical lines indicate the FC threshold of ±1, and the horizontal dashed line indicates the significance cutoff. (c) Bar chart showing passive tension measured in 3D muscle constructs at Day 8 (*n* = 8) and Day 21 (*n* = 12) of differentiation. Data represents mean ± standard deviation (SD). (d) Bar chart showing peak contractile force measured following electrical stimulation of 3D muscle constructs at Day 8 (*n* = 7) and Day 21 (*n* = 12) of differentiation. Data represents mean ± SD. (e) Bar chart showing the construct cross‐sectional area (CSA) at Day 8 (*n* = 4) and Day 21 (*n* = 5) of differentiation. Data represents mean ± SD. (f) Bar chart showing the effective CSA of constructs, taken as the area of the construct CSA positively stained for phalloidin to represent cellular content. Data represents mean ± SD. (g) Bar chart showing percentage of the cross‐sectional area positively stained with phalloidin, representing cellular content of 3D muscle constructs at Day 8 (*n* = 4) and Day 21 (*n* = 5) of differentiation. Data represents mean ± SD.

When comparing the 3D‐Day8 group to the myoblasts, 1019 DAPs were identified, consisting of 616 proteins showing increased abundance and 403 proteins showing decreased abundance in the 3D‐Day8 group. Unlike the mixed MHC isoform profile observed in 2D‐cultured muscle cells, those grown in 3D muscle constructs predominantly exhibited a slow‐twitch (type I) MHC 7 phenotype (Figure 6a).

Similar to the changes observed in the muscle cells cultured in 2D at the same timepoints, a large proportion of the up‐regulated proteins were associated with muscle development, with changes to proteins related to the cytoskeleton, muscle contraction, and the ECM. This was also reflected by the KEGG pathway analysis (Figure 5d). In particular, there was an increased abundance of all three subunits of laminin, including laminin alpha‐2, which is present in the basement membrane of mature muscle cells, and collagen IV chains, suggesting deposition of ECM components by the muscle cells in 3D culture (Gawlik & Durbeej, 2011; Khoshnoodi et al., 2008). Several isoforms of integrin alpha and beta chains were down‐regulated in the 3D‐Day8 group compared with myoblasts, potentially reflecting the change in adherence requirements between the actively proliferating myoblasts on a 2D culture surface and the more stable myotubes surrounded by a 3D hydrogel, as well as differences in ECM adhesion proteins present in both culture environments (Figure 6a).

The comparison with the fewest DAPs identified across the dataset was between 3D‐Day8 and 3D‐Day21 (65 DAPs), suggesting that the majority of myotube differentiation in 3D hydrogel culture had occurred by Day 8 in differentiation medium, similar to the results seen in 2D monolayer culture (Figure 6b). Noteworthy from the 65 DAPs identified, fast‐twitch myosin‐2 and the fast isoform of troponin T showed significantly increased abundance in 3D‐Day21 than 3D‐Day8, suggesting a shift towards a fast‐twitch phenotype with prolonged culture time in 3D. As predicted, given the limited number of DAPs between 3D‐Day8 and 3D‐Day21, the IPA analysis returned only a small number of enriched canonical pathways in this comparison, identifying downregulation of “mitochondrial dysfunction” (i.e., increased function) due to the increased abundance of cytochrome C oxidase subunit 7A1 in 3D‐Day21, suggesting potential improvements in mitochondrial function with longer differentiation duration (Table 2).

**TABLE 2:** Top up‐ and down‐regulated canonical pathways of human skeletal muscle cells differentiated in 3D hydrogels for 8 versus 21 days.

	Canonical pathway	*z*‐score	Ratio	−Log(*p*‐value)	Proteins present in dataset
Up‐regulated	Eicosanoid signaling	1.00	0.01	2.17	↑AKR1C3, ↑HSPB7, ↑PLCD3, ↓PTGDS
	Serotonin receptor signaling	1.00	0.01	1.43	↓F13A1, ↑MAOA, ↑PLCD3, ↑SPR
	Protein ubiquitination pathway	0.45	0.02	3.08	↓DNAJC1, ↑HSPA2, ↑HSPB6, ↑HSPB7, ↓IFT25
Down‐regulated	Mitochondrial dysfunction	−0.45	0.01	2.64	↑ATP1A2, ↑COX7A1, ↑MAOA, ↑MGST2, ↑SOD2

*Note*: Top up‐ and down‐regulated canonical pathways, ranked based on *z*‐score, identified by IPA analysis. For each pathway, the *z*‐score, ratio (proportion of proteins involved in the pathway), and −log(*p*‐value) are provided. The list of proteins from the dataset contributing to the differential expression of each pathway is included, with proteins showing increased abundance represented with up‐arrows and proteins showing decreased abundance represented with down‐arrows.

### 3.3 Effects of extended culture of 3D muscle constructs on structure and contractile force production

Functional assessment of 3D muscle constructs revealed a more than 20% increase in passive tension after 21 days of differentiation compared with Day 8 (Figure 6c). This was associated with a significant reduction in contractile force generation of 60% at 21 days post‐differentiation compared with data observed at Day 8 (Figure 6d). Values for passive tension measurements were considerably higher than those for contractile force generation.

**FIGURE 7:**
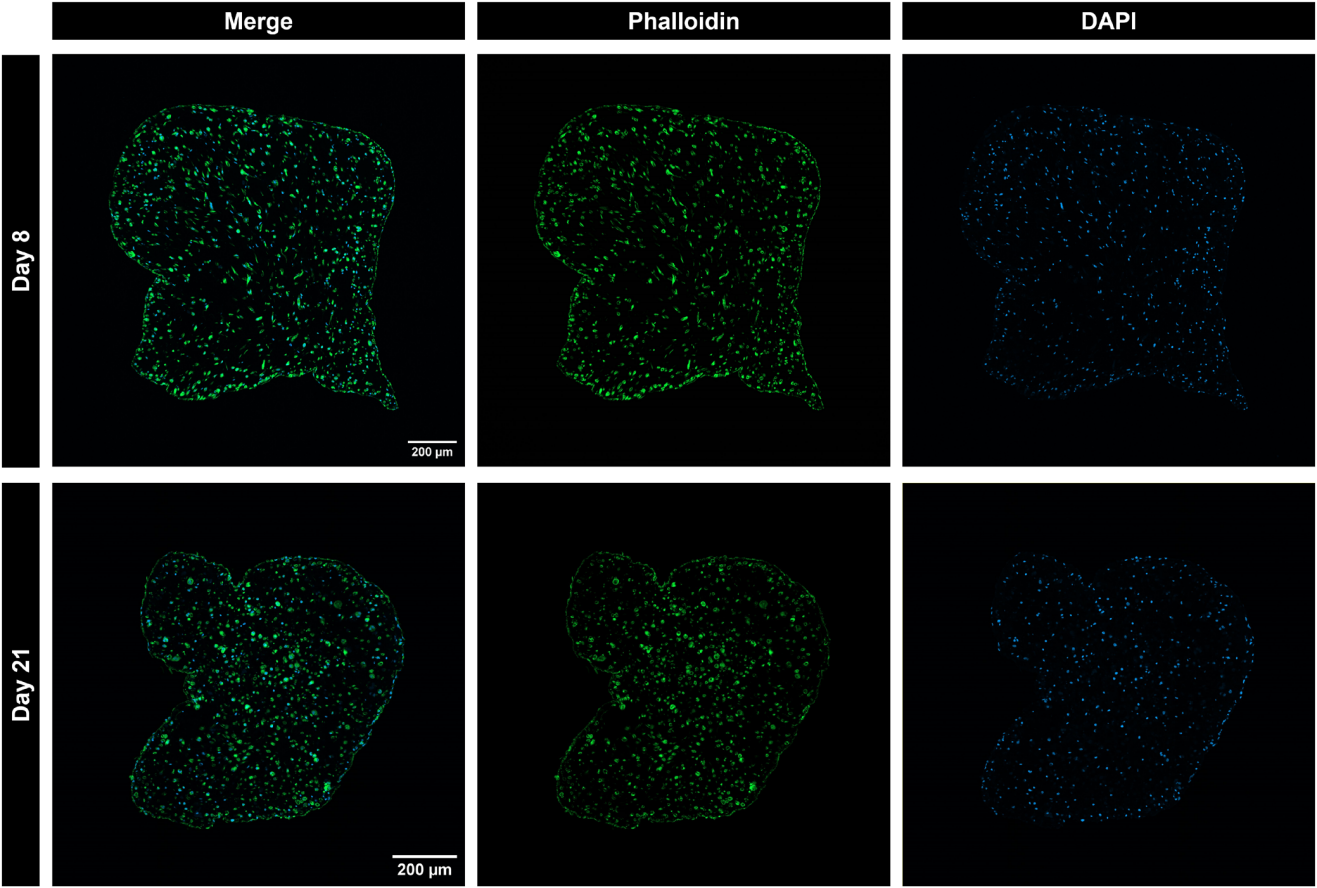
Immunofluorescence staining of human muscle construct cryosections. Representative images of transverse cryosections from human skeletal muscle cells cultured in 3D hydrogels at 8 or 21 days following initiation of differentiation. Cryosections were stained with phalloidin to identify the cellular content and DAPI for nuclear staining. Images were captured at 20× magnification. Scale bar 200 μm.

Immunofluorescent staining of cryosections from 3D muscle constructs revealed a significant reduction in construct CSA between Day 8 and 21 of differentiation (Figure 6e). However, the effective CSA, defined as the CSA of the muscle within the construct positively stained with phalloidin to represent muscle cell content, showed no significant difference, measuring 0.20 ± 0.034 mm^2^ at Day 8 and 0.16 ± 0.014 mm^2^ at Day 21 (Figure 6f). This stability in effective CSA resulted in an increase to the percentage of construct CSA represented by cells, and therefore myotube‐to‐hydrogel ratio, at Day 21 (Figure 6f). Based on these effective CSA values, the estimated specific force of the 3D muscle constructs decreased from 1.03 mN/mm^2^ at Day 8 to 0.74 mN/mm^2^. Representative cryosection images are presented in Figure 7.

### 3.4 Comparison of human skeletal muscle cells grown in 2D culture with 3D culture

When compared to undifferentiated myoblasts, the myotubes differentiated in 3D for 8 days resulted in a much larger change in the proteomic profile than myotubes in 2D culture for 8 days of differentiation, with more than twice as many DAPs identified between myoblasts and 3D‐Day8 (1019 DAPs) than between myoblasts and 2D‐Day8 (475 DAPs). Conversely, when considering the effects of a longer differentiation duration from 8 to 21 days, myotubes in 2D culture displayed over five times the number of DAPs (332 DAPs) than myotubes in 3D culture (65 DAPs) (Figure 2b). Together, these data suggest that muscle cells cultured in 3D have reached a stable point of differentiation after 8 days in differentiation medium, whereas 2D monolayers remain more dynamic in their proteomic profiles even after 8 days of differentiation.

**FIGURE 8:**
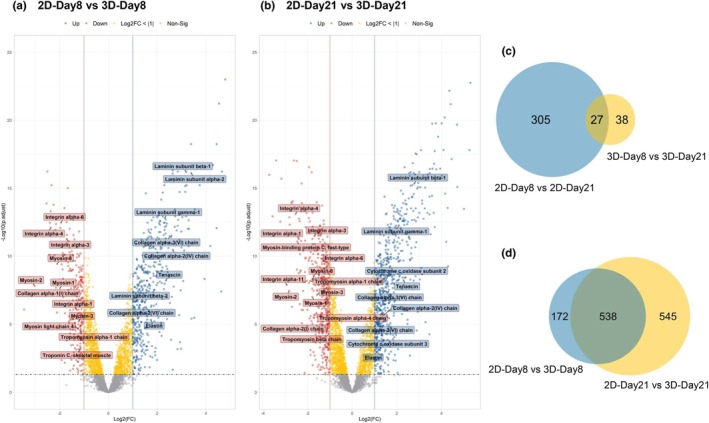
Differential protein abundance of human skeletal muscle cells cultured in 2D monolayers versus 3D hydrogels. (a, b) Volcano plots showing changes in protein abundance of human skeletal muscle cells cultured in 2D monolayers compared to 3D hydrogels, showing log2 fold change (FC) against –log10 adjusted *p*‐value. Differentially abundant proteins (DAPs) are defined by adjusted *p*‐value <0.05 and log2(FC) > |1|. DAPs are shown in blue (increased abundance) and red (decreased abundance). Proteins with a *p*‐value <0.05 but log2(FC) < |1| are shown in yellow and gray points represent non‐significant proteins. Vertical lines indicate the FC threshold of ±1, and the horizontal dashed line indicates the significance cutoff. (c) Venn diagram comparing the DAPs in human skeletal muscle cells following 8 or 21 days of differentiation in 2D monolayers (blue circle) or 3D hydrogels (yellow circle). The overlap shows 27 proteins (25 increased, 2 decreased) had differential abundance after extended culture in both 2D and 3D conditions. (d) Venn diagram comparing the DAPs between human skeletal muscle cells cultured in 2D or 3D following differentiation periods of 8 days (blue circle) and 21 days (yellow circle). The overlap shows 538 proteins (369 increased, 169 decreased) had differential abundance in 3D conditions compared to 2D conditions at both timepoints.

A total of 700 DAPs were identified when comparing 3D‐Day8 to 2D‐Day8, with 446 up‐regulated and 254 down‐regulated (Figure 2b). These are represented by the volcano plot in Figure 8a, where 3D samples show reduced abundance of several isoforms of myosin heavy chain and other key contractile proteins, suggesting a lower capacity for contraction in muscle cells grown in 2D. Conversely, proteins related to the ECM, such as several collagen and laminin isoforms, were higher in abundance in the 3D samples, suggesting remodeling of the surrounding hydrogel and deposition of ECM components by the myotubes in 3D culture.

This pattern of differential protein abundance was maintained when comparing 3D‐Day21 to 2D‐Day21, with additional contractile proteins, such as tropomyosin and myosin light chain, showing lower abundance in 3D culture (Figure 8b). Interestingly, 3D samples at Day 21 display significant upregulation of cytochrome C oxidase subunits compared to 2D samples at the same time points, suggesting improved mitochondrial function and metabolic activity of myotubes in 3D culture, potentially due to the more physiologically relevant architecture of the 3D hydrogel. KEGG analysis also reflected this shift in energy demands, particularly in glycolysis and pyruvate metabolism, between 2D and 3D cultures (Figure 5f,g).

The Venn diagram depicted in Figure 8c shows a total of 27 proteins that were identified in both the 2D‐Day8 versus 2D‐Day21 and 3D‐Day8 versus 3D‐Day21 comparisons, meaning 27 proteins were consistently differentially abundant with prolonged differentiation time. From these 27 proteins, 25 showed the same direction of change across both comparisons, with 24 proteins of higher abundance and 1 of lower abundance by 21 days of differentiation in either 2D or 3D culture. Notable among these was the increased abundance of the fast‐twitch isoform of troponin T and fast‐twitch myosin 2, suggesting myotubes shift towards a more fast‐twitch phenotype with prolonged culture duration in both growth conditions. Additionally, mitochondrial superoxide dismutase and cytochrome C oxidase subunit 7A1 were increased in abundance at 21 days of differentiation, indicating enhanced mitochondrial function and increased metabolic activity at later time points, possibly to account for increased energy demands or management of reactive oxygen species (ROS), or due to a reduced number of actively proliferating myoblasts relying on glycolytic metabolism.

As depicted in the Venn diagram in Figure 8d, 538 proteins were consistently differentially abundant in the 2D‐Day8 versus 3D‐Day8 and 2D‐Day21 versus 3D‐Day21 comparisons, meaning 538 proteins were consistently differentially abundant in 3D culture compared to 2D culture regardless of differentiation duration. The direction of change in abundance for all proteins was the same in both comparisons, with 369 increased and 169 decreased in abundance. Up‐regulated proteins across both timepoints included those involved in mitochondrial protein synthesis, glycolysis, oxidative stress response, and protein ubiquitination, suggesting 3D culture promotes mitochondrial function and cellular metabolic maturity. The DAPs that were consistently down‐regulated in the 3D cultures were primarily related to the cytoskeleton, muscle contraction, and cell growth. Notably, collagen I, the most common isoform of collagen found in native skeletal muscle, was down‐regulated in 3D samples at both timepoints (Hennessy & Simms, 2023).

IPA analysis comparing 2D and 3D cultures revealed consistent enrichment of canonical pathways related to energy metabolism, particularly glycolysis, in 3D samples. Additionally, 3D‐Day8 samples showed upregulation of reactive oxygen species detoxification systems compared to 2D‐Day8 samples (Table 3). Striated muscle contraction and cholesterol biosynthesis were among the top five most significantly down‐regulated canonical pathways in 3D cultures at both time points (Tables 3 and 4).

**TABLE 3:** Top up‐ and down‐regulated canonical pathways of human skeletal muscle cells cultured in 3D hydrogels versus 2D monolayers at Day 8 of differentiation.

	Canonical pathway	*z*‐score	Ratio	−Log(*p*‐value)	Proteins present in dataset
Up‐regulated	Neutrophil degranulation	4.26	0.11	16.20	↑ALAD, ↑ALDOA, ↑ANPEP, ↑APEH, ↑APRT, ↑ARPC5, ↓ASAH1, ↓CD44, ↓CD47, ↑COTL1, ↑CPNE1, ↑CPNE3, ↑CPPED1, ↑CST3, ↑CSTB, ↓CTSC, ↑FABP5, ↑FTH1, ↑GAA, ↑GDI2, ↑GM2A, ↑GPI, ↑GSN, ↑GSTP1, ↑HEBP2, ↓LAMP1, ↑LTA4H, ↑MIF, ↑MME, ↑NAPRT, ↓NEU1, ↑NIT2, ↑NME2, ↑PDXK, ↑PFKL, ↑PGM1, ↑PGM2, ↑PKM, ↓PLAU, ↑PPIA, ↑PRDX6, ↑PTX3, ↑PYGB, ↑PYGL, ↓RAB5C, ↑S100A11, ↑SERPINB1, ↑SERPINB6, ↑SLC2A5, ↓STING1, ↓STK10, ↓TCIRG1, ↑TMT1A
	Glycolysis I	3.61	0.48	12.90	↑ALDOA, ↑ENO1, ↑ENO3, ↑GAPDH, ↑GPI, ↑PFKL, ↑PFKM, ↑PFKP, ↑PGAM2, ↑PGK1, ↑PGK2, ↑PKM, ↑TPI1
	Detoxification of reactive oxygen species	3.46	0.32	9.48	↑ATOX1, ↑CCS, ↑ERO1A, ↑GPX1, ↑GSR, ↑GSTP1, ↑NUDT2, ↑PRDX1, ↑PRDX2, ↑PRDX6, ↑SOD1, ↑SOD2
	Gluconeogenesis I	3.16	0.37	8.64	↑ALDOA, ↑ENO1, ↑ENO3, ↑GAPDH, ↑GPI, ↑MDH1, ↑ME1, ↑PGAM2, ↑PGK1, ↑PGK2
	Glycogen degradation III	3.00	0.69	11.00	↑GAA, ↑MTAP, ↑PGM1, ↑PGM2, ↑PGM3, ↑PYGB, ↑PYGL, ↑PYGM, ↑TYMP
Down‐regulated	Striated muscle contraction	−2.83	0.22	5.13	↓MYH3, ↓MYH6, ↓MYH8, ↓MYL1, ↓MYL4, ↓TNNC2, **↓**TNNT2, ↓TPM1
	Cholesterol biosynthesis I	−2.65	0.54	7.59	↓DHCR7, ↓FDFT1, ↓HSD17B7, ↓LSS, ↓MSMO1, ↓NSDHL, ↓SQLE
	Cholesterol biosynthesis II (via 24,25‐dihydrolanosterol)	−2.65	0.54	7.59	↓DHCR7, ↓FDFT1, ↓HSD17B7, ↓LSS, ↓MSMO1, ↓NSDHL, ↓SQLE
	Cholesterol biosynthesis III (via desmosterol)	−2.65	0.54	7.59	↓DHCR7, ↓FDFT1, ↓HSD17B7, ↓LSS, ↓MSMO1, ↓NSDHL, ↓SQLE
	SNARE signaling pathway	−2.53	0.07	2.17	↓GOSR2, ↓MYH1, ↓MYH2, ↓MYH3, ↓MYH6, ↓MYH8, ↓MYL1, ↓MYL4, ↑PRKACA, ↓VAMP3

*Note*: Top 5 up‐ and down‐regulated canonical pathways, ranked based on *z*‐score, identified by IPA analysis. For each pathway, the *z*‐score, ratio (proportion of proteins involved in the pathway), and −log(*p*‐value) are provided. The list of proteins from the dataset contributing to the differential expression of each pathway is included, with proteins showing increased abundance represented with up‐arrows and proteins showing decreased abundance represented with down‐arrows.

**TABLE 4:** Top up‐ and down‐regulated canonical pathways of human skeletal muscle cells cultured in 3D hydrogels versus 2D monolayers at Day 21 of differentiation.

	Canonical pathway	*z*‐score	Ratio	−Log(*p*‐value)	Proteins present in dataset
Up‐regulated	Glycolysis I	3.742	0.519	11.9	↑ALDOA, ↑ENO1, ↑ENO3, ↑GAPDH, ↑GPI, ↑PFKL, ↑PFKM, ↑PFKP, ↑PGAM2, ↑PGK1, ↑PGK2, ↑PKLR, ↑PKM, ↑TPI1
	TP53 Regulates metabolic genes	3.5	0.182	5.83	↑COX5B, ↑COX6B1, ↑COX6C, ↑COX7B, ↓GLS, ↑GPI, ↑GSR, ↑MT‐CO3, ↑NDUFA4, ↑PRDX1, ↑PRDX2, ↑RRM2B, ↑TIGAR, ↑TXN, ↑TXNRD1, ↑YWHAH
	Glycogen metabolism	3.317	0.458	8.73	↑AGL, ↑GAA, ↑GBE1, ↑GYS1, ↑PHKA1, ↑PHKB, ↑PHKG1, ↑PYGB, ↑PYGL, ↑PYGM, ↑UGP2
	Protein folding	3.3	0.184	6.55	↑ARFGEF2, ↑CCT2, ↑CCT4, ↑CCT5, ↑CCT6A, ↑CCT6B, ↑CCT7, ↑CCT8, ↓FKBP9, ↓GBA1, ↑PFDN1, ↑PFDN5, ↑TBCA, ↑TBCB, ↑TBCD, ↑TBCE, ↑TCP1, ↑TUBB6
	Class I MHC mediated antigen processing and presentation	3.266	0.064	1.32	↓BCAP31, ↑BLMH, ↑FBXL8, ↑FBXO22, ↑FBXO30, ↑HECTD1, ↑HECTD3, ↓HLA‐A, ↑HUWE1, ↑IKBKB, ↑NPEPPS, ↑PSMD5, ↑PSMD9, ↑PSME2, ↑PSME4, ↑RNF123, ↓TAPBP, ↑THOP1, ↑TPP2, ↑UBA1, ↑UBA6, ↑UBAC1, ↑UBE2O, ↓VAMP3
Down‐regulated	ILK signaling	−3.606	0.0746	1.45	↓ACTN4, ↑FLNB, ↓FN1, ↑GSK3A, ↑LIMS1, ↓MYH1, ↓MYH2, ↓MYH3, ↓MYH6, ↓MYH8, ↓MYH9, ↓MYL1, ↓MYL4, ↑PPP2R1A, ↑RSU1
	Striated muscle contraction	−3.464	0.333	7.58	↓DES, ↓MYBPC2, ↓MYH3, ↓MYH6, ↓MYH8, ↓MYL1, ↓MYL4, ↓TNNC2, ↓TNNT1, ↓TPM1, ↓TPM2, ↓TPM4
	SNARE signaling pathway	−3.464	0.0882	1.74	↓GOSR2, ↓MYH1, ↓MYH2, ↓MYH3, ↓MYH6, ↓MYH8, ↓MYH9, ↓MYL1, ↓MYL4, ↓RAB6A, ↓VAMP3, ↓VTI1B
	Cholesterol biosynthesis I	−2.828	0.615	7.8	↓DHCR24, ↓DHCR7, ↓EBP, ↓FDFT1, ↓HSD17B7, ↓LSS, ↓NSDHL, ↓SQLE
	Cholesterol biosynthesis II (via 24,25‐dihydrolanosterol)	−2.828	0.615	7.8	↓DHCR24, ↓DHCR7, ↓EBP, ↓FDFT1, ↓HSD17B7, ↓LSS, ↓NSDHL, ↓SQLE

*Note*: Top 5 up‐ and down‐regulated canonical pathways, ranked based on *z*‐score, identified by IPA analysis. For each pathway, the *z*‐score, ratio (proportion of proteins involved in the pathway), and −log(*p*‐value) are provided. The list of proteins from the dataset contributing to the differential expression of each pathway is included, with proteins showing increased abundance represented with up‐arrows and proteins showing decreased abundance represented with down‐arrows.

## 4 DISCUSSION

This study provides novel insights into the differentiation, maturation, and functional characteristics of human skeletal muscle cells cultured in both 2D and 3D environments. A human skeletal muscle cell line was cultured in either 2D monolayers or embedded within a fibrin‐based hydrogel 3D environment as a muscle construct, and samples were analyzed at key timepoints throughout differentiation for a total of 21 days. Proteomic analysis was paired with functional measures of muscle constructs to determine whether a 3D environment or extended culture duration promotes the maturity of skeletal muscle cells in vitro.

In both 2D and 3D environments, myoblasts successfully differentiated into myotubes by Day 8 of differentiation, as shown by the presence of key contractile proteins. Culturing muscle cells in 2D monolayers resulted in a distinct proteomic profile compared with muscle cells cultured in a 3D hydrogel, highlighting the potential importance of considering culture environment and its influence on the cellular phenotype when selecting and developing in vitro cell culture models.

When comparing the proteomic profiles of muscle cells grown in 2D or 3D culture over time, muscle cells in 3D culture appeared to have stabilized by Day 8 of differentiation, with minimal changes to the protein abundances observed between the 3D‐Day8 and 3D‐Day21 groups. In contrast, muscle cells in 2D culture exhibited greater differences in their protein abundances between 2D‐Day8 and 2D‐Day21, potentially reflecting either a more dynamic proteomic profile or a delayed and prolonged differentiation process in 2D culture.

Changes in the abundance patterns of myosin heavy chain (MHC) isoforms between 2D cultured and 3D cultured muscle cells also provided insight into the effects the culture environment has on the differentiation and maturity of myotubes. Consistent with a previous study of 3D bioprinted human skeletal muscle, culture of muscle cells in 3D environments promoted a predominantly slow‐twitch (type I) phenotype, likely associated with the sustained, low‐intensity contraction required by the myotubes to maintain structural support when not cultured on rigid 2D culture surfaces (Alave Reyes‐Furrer et al., 2021). The increase in abundance of oxidative fast‐twitch (type IIa) MHC 2 with prolonged culture in both 2D and 3D environments, as well as the upregulation of oxidative phosphorylation pathways and improved mitochondrial maturity observed in the proteomic dataset, correlates with the known MHC expression patterns observed in native human skeletal muscle during development (Chal & Pourquié, 2017; Schiaffino & Reggiani, 1985). This indicates that the myotubes were potentially in the early stages of transitioning towards a more oxidative, fast‐twitch phenotype with prolonged culture, reflecting sarcomere maturation seen in previous studies (Afshar Bakooshli et al., 2019). The greater abundance of slow‐twitch MHC isotypes over fast‐twitch isotypes in 3D cultured muscle cells presented here is in agreement with previously published data using primary human muscle cells, validating the use of the immortalized cell line for the generation of 3D muscle constructs as an alternative to primary cell cultures, while also providing an extensive proteomic dataset (Alave Reyes‐Furrer et al., 2021; Dalmao‐Fernandez et al., 2023). The novelty of the present study lies in the integration of the observations to functional measures.

Although the overall abundance of MHC was lower in muscle cells cultured in 3D compared to 2D, many key human extracellular matrix (ECM) proteins were more abundant in 3D muscle constructs. Alongside the protein indices of elevated energy metabolism observed in 3D samples, this suggests that muscle cells within 3D hydrogels directed energy and resources towards an ECM remodeling phase to adapt to their surrounding environment, rather than prioritizing contraction, and highlights the high energy demands associated with ECM deposition and degradation (Guillard & Schwörer, 2024; Sthijns et al., 2021). Data presented here suggest that culturing muscle cells in 3D more closely resembles the complex, ECM‐rich environment of native skeletal muscle and produces a physiologically relevant balance of biochemical cues, energy demands, and cellular functions observed in native muscle (Ahmad et al., 2023).

These effects of ECM remodeling are also observed in the reduced CSA of muscle constructs accompanied by the increased cell‐to‐hydrogel ratio between Day 8 and 21 of differentiation. This change to the muscle construct structure enhances cell‐to‐cell interactions and coupling, potentially leading to the observed increase in passive tension at the later time point. Consequently, this increased passive tension may create a greater resistance to contraction during electrical stimulation, contributing to the reduction in measured contractile force at Day 21. Since the effective CSA of the muscle constructs, and therefore the quantity of cellular content, remained consistent between Day 8 and 21, the other potential factor driving the loss of contractile force at the later time point is a reduction in the quality of myotubes. When studying bioengineered skeletal muscle models, the ability to determine specific force values when accounting for the size and amount of muscle present within the construct at different times is invaluable. Specific force estimations calculated in this study are within the typical range for bioengineered muscle models, comparable to those using both primary cells and iPSCs. Data presented here support the hypothesis that prolonged culture of muscle constructs resulted in a reduced contractile force capability of myotubes (Khodabukus et al., 2018; Rao et al., 2018; Rose et al., 2023; van der Wal et al., 2023; Vesga‐Castro et al., 2022).

A limitation of the present study that should be noted is the inherent variation in bioengineered tissue formation, particularly when using naturally derived components prone to batch‐to‐batch variability, such as Matrigel and fibrinogen. Exploring synthetic alternatives to Matrigel could provide a more consistent and modifiable scaffold for culturing muscle cells in 3D environments. Additionally, myoblast samples used in this study were harvested during the active proliferation phase, the proteomes of which may vary from quiescent myoblasts (satellite cells) found in mature native skeletal muscle (Chal & Pourquié, 2017). In the current study, analysis was limited to proteotypic proteins with confirmed human origin, narrowing the scope of the proteomic profile examined. Developing methods to appropriately incorporate non‐proteotypic proteins would allow for the inclusion of proteins which share sequence homology with the species of origin of the hydrogel components. Relative levels of myoblasts compared with myotubes present in the preparation should also be taken into consideration when comparing proteomic differences, particularly between 2D timepoints where partial cell detachment is possible (such as in the early stages of differentiation in 2D in this preparation) and this is an additional limitation to 2D analyses.

Other potential future studies could focus on extended culture times of 3D muscle constructs, beyond the 21 days of differentiation used in the present study, to explore how the transition towards a more actively contractile, fast‐twitch phenotype continues. Additionally, it would be worthwhile to investigate whether this transition, combined with the potential redirection of energy and resources towards developing contractile machinery after the cells have adapted to and remodeled the 3D environment, leads to improved contractile forces. Proteomic comparisons of muscle constructs beyond 21 days of differentiation or following innervation (a process known to result in further maturation in vitro) to native human muscle tissue would also be of interest for placement of 3D muscle constructs on the continuum between 2D monolayers and native tissue. Evidence suggests that a limiting factor in muscle maturity during myogenesis is innervation of muscle fibers and so further development of this model to incorporate innervation in a co‐culture is warranted (Afshar Bakooshli et al., 2019; Kim et al., 2020; Maggs et al., 2008; Saini et al., 2021). Beyond this, functional measures of changes in energy metabolism would also be of interest for future work to compare to the proteomic dataset provided in the present study. The AB1167 cell line offers a major step forward in translational research, primarily due to the general lack of availability of immortalized human muscle cell lines compared with the commonly used mouse C2C12 cell line. It would be of interest to expand the proteomic characterization of the effects of culture conditions to include alternative immortalized human muscle cell lines, derived from both healthy control donors and those of different ages or with varying musculoskeletal diseases, but such extensive comparisons are beyond the scope of the current study (Mamchaoui et al., 2011).

In conclusion, the data presented reveal that both culture environments (2D or 3D) and duration of culture significantly influence the proteomic profiles, functional properties, and maturation of human skeletal muscle cells. Although 2D and 3D systems each offer unique advantages and challenges for different experimental needs, the results suggest that 3D culture promotes signs of metabolic maturity, and the peak contractile force at Day 8 of differentiation makes this system well‐suited for studying musculoskeletal disorders and testing therapeutic interventions. By highlighting the importance of consideration and refinement of culture conditions to balance functional performance and physiological relevance to mature native skeletal muscle, these findings can help guide future attempts at bioengineering an improved in vitro model of human skeletal muscle.

## AUTHOR CONTRIBUTIONS

Benjamin R. Tollitt and Anne McArdle conceived and designed research. Jessica Ohana provided cell line. Benjamin R. Tollitt performed experiments. Benjamin R. Tollitt analyzed data. Benjamin R. Tollitt, Samantha W. Jones, Malcolm J. Jackson, and Anne McArdle interpreted results of experiments. Benjamin R. Tollitt prepared figures. Benjamin R. Tollitt drafted manuscript. Benjamin R. Tollitt, Samantha W. Jones, Malcolm J. Jackson, and Anne McArdle edited and revised manuscript. All authors approved final version of the manuscript.

## FUNDING INFORMATION

UKRI Medical Research Council, DiMeN Doctoral Training Partnership, Grant/Award Number: MR/N013840/1 (McArdle). UKRI Biotechnology and Biological Sciences Research Council, Grant/Award Number: BB/X51200X/1 (McArdle).

## CONFLICT OF INTEREST STATEMENT

The authors declare that they have no competing interests.

## ETHICS STATEMENT

Ethics approval and consent to participate is not applicable. The human‐derived immortalised cell line, previously immortalised by Vincent Mouly laboratory were provided by biobank MyoLine and used according to a Material Transfer Agreement.

## Data Availability

The mass spectrometry proteomics data have been deposited to the ProteomeXchange Consortium via the PRIDE (Perez‐Riverol et al., 2025) partner repository with the dataset identifier PXD061227.
